# Retinopathy of prematurity: from oxygen management to molecular manipulation

**DOI:** 10.1186/s40348-023-00163-5

**Published:** 2023-09-15

**Authors:** Jonathan Woods, Susmito Biswas

**Affiliations:** 1 University of Birmingham Medical School, Medical School, College of Medical and Dental Sciences, University of Birmingham, Edgbaston, Birmingham, B15 2TT UK; 2 Manchester Royal Eye Hospital, Manchester University Hospital NHS Foundation Trust, Oxford Rd, Manchester, M13 9WL UK

**Keywords:** Infant, Newborn, Retinopathy of prematurity, Ophthalmoscopy, Cell proliferation, Oxygen

## Abstract

**Introduction:**

Retinopathy of prematurity (ROP) is a vasoproliferative disorder of the premature retina with the potential to progress to extraretinal neovascularisation. This review serves as an introduction to retinopathy of prematurity (ROP), outlining key parts of ROP pathophysiology, diagnosis and treatment. ROP is traditionally diagnosed by indirect ophthalmoscopy and classified using anatomical zones, stages of disease, and the presence or absence of “plus disease” (dilation and tortuosity of the major retinal arterioles and venules). ROP has a bi-phasic pathophysiology: initial hyperoxia causes reduced retinal vascularisation, followed by pathological vaso-proliferation resulting from subsequent hypoxia and driven by vascular endothelial growth factor (VEGF).

**Advancements in management:**

This review summarises previous trials to establish optimum oxygen exposure levels in newborns and more recently the development of anti-VEGF agents locally delivered to block pathological neovascularisation, which is technically easier to administer and less destructive than laser treatment.

**Future directions:**

There remains an ongoing concern regarding the potential unwanted systemic effects of intravitreally administered anti-VEGF on the overall development of the premature baby. Ongoing dosing studies may lessen these fears by identifying the minimally effective dose required to block extraretinal neovascularisation.

## Introduction

Preterm birth has a potentially wide-ranging impact on neurovascular development, including the neonatal retina. The incidence of premature birth is increasing globally and varies internationally (5–18% in some European and African states, respectively) [1]. Preterm survival is also increasing globally; this progress is more marked in countries with rapidly improving neonatal facilities [2] that are sufficiently developed to increase preterm survival, but insufficiently developed to reduce associated morbidity [3]. There is, therefore, an increasing number of preterm babies at risk of lifelong disability due to complications associated with prematurity.

Retinopathy of prematurity (ROP) is a vasoproliferative disorder of the premature retina with the potential to progress to extraretinal neovascularisation. This can subsequently lead to retinal detachment and sight loss. A recent multicentre audit in the UK revealed that 4% of babies with a birth weight (BW) < 1500 g required treatment for ROP [4]. The incidence of ROP in settings with less developed neonatal facilities is higher, since ROP can develop in babies of greater birth weight and gestational age (GA) in these settings [3, 5].

### Prematurity and normal eye development

#### Retinal vascular development

The retina consists of organised layers of photoreceptors, interneurons, glial elements, and their various interconnections, overlying the retinal pigment epithelial layer. The retina receives oxygen through a dual blood supply with the outer layers receiving a supply from the choroidal layer and the inner retina predominantly receiving a supply from the retinal vasculature. Vascularisation of the retina starts in the second month of gestation from the optic disc and moves peripherally behind a “wave of neuronal differentiation” and consequent increasing metabolic activity [6], reaching the nasal side in the eighth month and the temporal side at approximately the time of term delivery.

#### The role of oxygen in the pathogenesis of ROP

In utero, the partial pressure of oxygen (PO_2_) in the umbilical vein is less than 50 mmHg [7]. Thus, retinal vasculature develops in a controlled, relatively hypoxic environment. Following delivery, arterial PO_2_ rises; neonates given supplementary oxygen may be exposed to yet higher, more fluctuating levels of oxygen. Crosse and Jameson observed in 1952 that the incidence of ROP in neonates rose and fell with the liberal and restricted administration of oxygen, respectively [8]. The ELGAN study corroborates that such hyperoxia on blood gases contributes to the development of ROP [9]. Blood transfusion containing adult haemoglobin may further increase oxygen availability in developing structures due to the different oxygen affinities of foetal and adult haemoglobin [10]. The preterm retina is particularly vulnerable to hyperoxia for two reasons: the immature vasculature has a reduced ability to autoregulate [11], and the reserve of antioxidants is not large enough to protect from damage by reactive oxygen species [12] due to prevention of full transfer of $\varpi$-long-chain polyunsaturated fatty acids (LCPUFAs) from mother to baby in the third trimester [13]. Limited data from clinical trials indicate the potential for supplementation of LCPUFAs in the early post-natal period may reduce the incidence of severe ROP [14–16].

#### Levels of insulin-like growth factor-1 (IGF-1) in the premature neonate

Reduced concentrations of IGF-1, seen in preterm neonates [17], is a key contributor to ROP development due to its synergistic action with VEGF where angiogenesis occurs only when IGF-1 reaches a critical level [18]. The deficit in IGF-1 means that vitreoretinal VEGF levels continue to build until IGF-1 reaches the critical level to support angiogenesis. The supranormal levels of VEGF then result in extraretinal neovascularisation [19]. The possibility that supplemental IGF-1 given from 24 h of birth until reaching 30 weeks postmenstrual age (PMA) might prevent severe ROP was tested in a clinical trial with intravenous infusions of supplemental recombinant human IGF-1 combined with recombinant human IGF-1 binding protein. Unfortunately, no reduction in severe ROP was observed in the treatment arm compared to the control arm although there was a reduced incidence of bronchopulmonary dysplasia in the treatment arm. Further studies are required to clarify the potential of IGF-1 [20].

#### Risk factors

Understanding retinal development in the context of prematurity provides an explanation for the risk factors associated with ROP: gestational age, birth weight, post-natal weight gain and nutrition linked to IGF-1 [21] and oxygen exposure [9]. Therefore, ROP may feature alongside other conditions associated with disrupted vascular development or abnormal fluctuations in oxygen saturation such as poor brain growth, bronchopulmonary dysplasia and necrotising enterocolitis.

Dysregulation of blood vessel development due to infection or inflammation, such as chorioamnionitis, may also form a “prephase” of ROP [22]. It is unknown whether intra-uterine events that may cause preterm delivery predispose to the development of ROP. Higher incidence in males and Caucasian babies suggests there may also be a genetic predisposition as implied by ethnic variations in incidence of severe ROP [23].

### Pathogenesis

Retinopathy of prematurity can be thought of in two phases: the cessation of normal vascular development in high-oxygen environments, followed by pathological compensatory vascularisation of the retina [24].

#### Phase one (hyperoxia)

In addition to decreased levels of IGF-1 and maternal $\omega$-LCPUFAs, exposure of the premature retina to increased oxygen causes further dysregulation of vascular development by suppression of hypoxia-induced factors (HIFs) which reduces levels of angiogenic factors such as vascular endothelial growth factor (VEGF) and erythropoietin (EPO) [25]. In extreme cases, an oxygen-induced retinopathy has been demonstrated in pre-term babies (≥ 28 weeks and ≥ 1000 g) given unmonitored and unblended 100% oxygen for 3 days or longer after birth [26].

#### Phase two (hypoxia)

As the retina develops, its metabolic demand increases such that the environment becomes hypoxic. In hypoxic conditions, the breakdown of HIFs is reduced [27, 28]. HIFs upregulate the expression of VEGF which acts via specific receptors (VEGFR-1 and VEGFR-2) [29] to cause increased migration, proliferation and permeability of endothelial cells. In ROP, this process becomes pathological due to the excessive amounts of VEGF in the absence of sufficient IGF-1 to support angiogenesis. Once IGF-1 reaches sufficient levels, the excess VEGF causes extraretinal neovascularisation which subsequently undergoes cicatrisation. This cicatricial process is accompanied by contracture of fibrous metaplastic tissue which, if extensive enough, can lead to a tractional retinal detachment [18, 30, 31]. Whilst the role of EPO in ROP is not well elucidated, excessive concentrations of EPO in the vitreous have also been correlated with levels of vitreous VEGF and partial retinal detachment [32].

#### Timing of phase transition

**Fig. 1:**
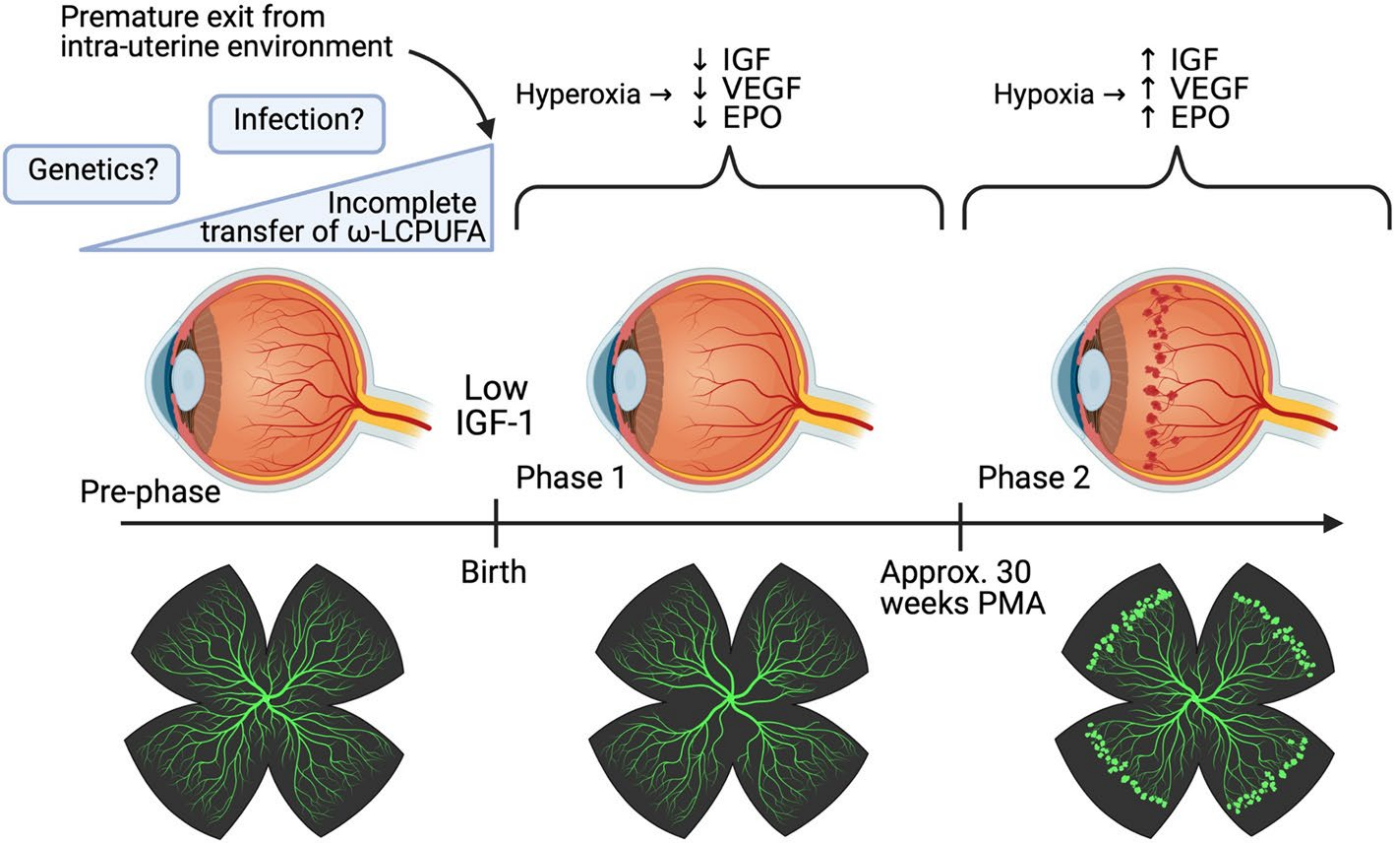
The 2-phase model of ROP. Diagrams shown below the timeline are cartoons of retinal flat mounts, similar to those seen in rat and mouse model

The transition from phase 1 to phase 2 has a significant bearing on the timing of initiation for screening of ROP, the purpose of which is to identify sight-threatening ROP before it becomes untreatable. Whilst this is not precisely known in any given premature baby, we know from natural history studies that there is a close association of ROP development to post-menstrual age [33]. In babies born before 27 weeks of gestation, the earliest onset of stage 3 ROP is not earlier than 31 weeks, and therefore, this can be used as a guide to initiate screening in the most premature neonates [34]. Figure 1 shows a summary diagram of the progression of ROP (Fig. 1).

### Diagnosis and classification

**Fig. 2:**
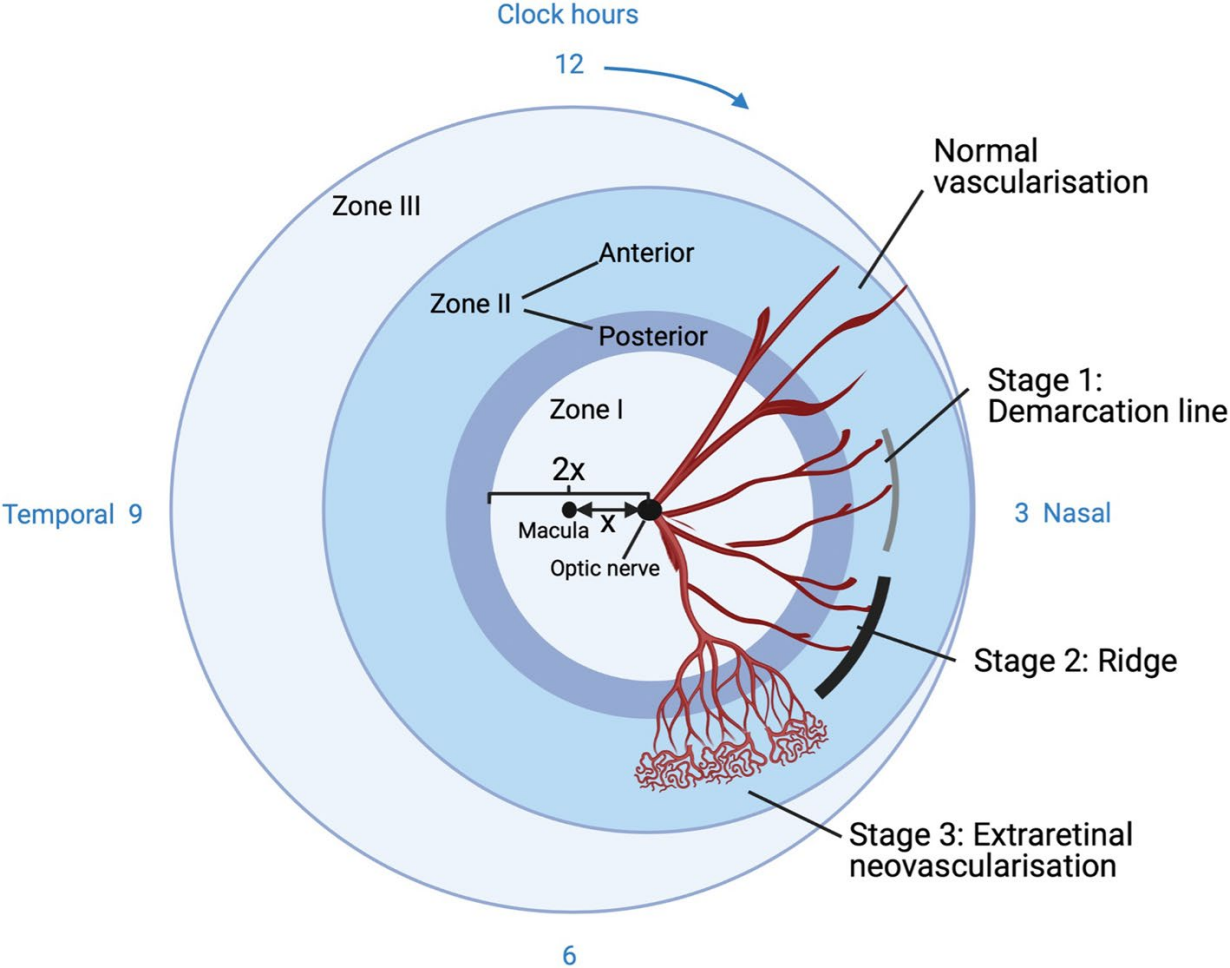
Zones of the right eye and appearance of retinal vasculature according to ROP stage. Zone I, the most posterior, is a circular zone centred on the optic nerve and with a radius twice the distance (2x) from the centre of the optic nerve to the centre of the macula (x). Zone II represents a middle zone, concentric to zone I, extending from the border of zone I to the nasal ora serrata (furthest extent of the nasal retina). Zone II is itself subdivided into a posterior zone II, two disc diameters out from the border of zone I, and anterior zone II encompassing the remaining peripheral area of zone II. Zone III is the most peripheral zone extending from the temporal border of zone II and capturing a remaining temporal crescentic area beyond zone II. ROP is likely to be more severe when located in zone I or posterior zone II. Stages 1–3 can occur in any zone of the eye. Here, they are shown in zone II for clarity

ROP is traditionally diagnosed by indirect ophthalmoscopy but, more recently, is increasingly being identified through digital retinal imaging [35]. ROP is classified using the International Classification of ROP (ICROP3) standards [36] which denote ROP in terms of zones, stages, clock hours (extent) and “plus disease”. The retina is divided into three concentric zones (Fig. 2). The stages are defined by the appearance of the retinal vasculature at the junction of the vascularised retina with the non-vascularised retina. Initially, this may appear as just incomplete vascularisation (immature vascularisation), previously termed stage 0. Stage 1 represents a pale demarcation line at the junction between vascularised and avascular retina, stage 2 represents an elevation of the demarcation line (ridge) and stage 3 represents the development of extraretinal neovascularisation. Small angioblastic buds, termed “popcorn”, may appear above the stage 2 ridge presaging the development of stage 3. Changes in the appearance of the blood vessels at the posterior pole within zone I may be noted during the progression of ROP with gradually increasing vessel dilation and tortuosity of the major arterioles and venules, termed “plus disease”. This is a significant driver for treatment for ROP and is therefore a critical feature. There is a gradation in the progression of vessel tortuosity and dilation from the absence of plus disease to plus disease with intermediate levels being termed “pre-plus”. Distinguishing plus disease from pre-plus is often a matter of judgement and there is considerable subjectivity and, consequently, interobserver variation, but is likely to be aided in the future with the use of artificial intelligence algorithms [37]. However, it is possible that the apparent size and shape of vessels become distorted by the pressure change associated with using contact wide-angle retinal imaging camera lenses [38]. Stage 3 ROP in the presence of plus disease forms a fibrovascular complex that may progress to a tractional retinal detachment. Where there is a partial detachment, this is termed stage 4: if not involving the macula, this is stage 4a and if involving the macula, this is stage 4b. Stage 5 denotes a total detachment of the retina and is further subdivided into stage 5a (total retinal detachment); stage 5b (total detachment of the retina with a closed funnel configuration); stage 5c (accompanying changes to the anterior segment of the eye such as cataract, glaucoma and corneal opacity). Stages 4 and 5 are typically associated with poor visual outcomes and blindness.

#### Implications for management

As ROP has two phases, there is a lag phase from birth to the development of the ROP lesion. A balance therefore needs to be struck between screening unnecessarily early and commencing in a timely way in order to identify preterm infants at risk of sight-threatening ROP. UK guidelines recommend that eye examinations are not carried out before 31 weeks PMA [39], due to observational studies showing that severe ROP is not seen before this point [34]. Accurate classification of ROP is not only important for identifying babies that require treatment, but is also for identifying infants that are at higher risk of progression and therefore requiring more frequent monitoring intervals. Table 1 shows how zone, stage and the presence of plus disease inform types of ROP. Treatment-warranted ROP is termed “type 1”. “Type 2” ROP represents ROP status that requires careful monitoring, on a weekly basis. Babies that have immature vascularisation only in anterior zone II, stage 1 in anterior zone II or stage 2 in anterior zone II without any plus or pre-plus disease are all deemed to be lower risk and may be monitored at two weekly intervals. Immature vascularisation terminating in zone I or posterior zone II; or stage 1 or 2 ROP in zone I or posterior zone II or stage 3 ROP in Zone II (type 2 ROP); the presence of any pre-plus disease, all increase the risk of progression and would necessitate more frequent, weekly monitoring [39].

**Table 1:** Using stage, zone, and presence of “plus disease” to classify ROP into type 1 and type 2

	Zone I		Zone II		Zone III
Stage 1	⊕ = ***Type* 1**	⊖ = ***Type* 2**	Examination intervals based on plus / pre-plus disease status		Examination intervals based on plus / pre-plus disease status
Stage 2	⊕ = ***Type* 1**	⊖ = ***Type* 2**	⊕ = ***Type* 1**	Examination intervals based on pre-plus disease status	Examination intervals based on plus / pre-plus disease status
Stage 3	⊕ = ***Type* 1**	⊖ = ***Type* 1**	⊕ = ***Type* 1**	⊖ = ***Type* 2**	Examination intervals based on plus / pre-plus disease status

⊕ Plus disease

⊖ No plus disease

Aggressive ROP (A-ROP) is a rare but severe form of ROP. This typically develops in zone I or posterior zone II, but may be located in more anterior zones in some parts of the world with limited resources available for optimal neonatal care. The hallmark of A-ROP is the marked prominence of plus disease typically in all 4 retinal quadrants of the eye with a poorly defined, flat neovascularisation not associated with a demarcation line or ridge.

### Prevention and treatment

#### Oxygen management

Between 1994 and 2014, large, multi-centre randomised control trials (RCTs)—STOP-ROP [40], SUPPORT [41], BOOST II [42], and COT [43]—have been conducted across developed countries. Table 2 compares these studies (Table 2).

**Table 2:** Summary of studies comparing different target SaO_2_ for prevention of ROP

	STOP-ROP		Support		Boost II		COT	
Location	30 centers in USA		22 centers in USA		Centers in Australia (*n *= 9), New Zealand (*n* = 5), and UK (*n *= 20)		25 centers in Canada, USA, Argentina, Finland, Germany, Israel	
Recruitment period	1994–1999		2005–2009		2006–2010		2006–2010	
Latest follow-up	3 months after due date		Discharge or 36 weeks (latest)		2 years		18 months	
Inclusion criteria	Preterm infants AND confirmed ROP in $\ge$ 1 eye AND median SaO_2_ < 94%		GA: 24–26 weeks + 6 days		GA < 28 weeks		GA: 23–27 weeks + 6 days	
Number of participants	649		1316		2448		1201	
Target oxygen saturations	89–94%	96–99%	85–89%	91–95%	85–89%	91–95%	85–89%	91–95%
Mortality	2.2%	2.8%	19.9%	16.2%	23.1%	15.9%	16.6%	15.3%
	No RR/OR available		RR = 1.27 (95% CI = 1.01–1.60)		RR = 1.45 (95% CI = 1.15–1.84)*		OR = 1.11 (95% CI = 0.80–1.54)	
Development of severe ROP	48%	41%	8.6%	17.9%	10.6%	13.5%	12.8%	13.1%
	OR = 0.72 (95% CI = 0.52–1.01)		RR = 0.52 (95% CI = 0.37–0.73)		RR = 0.79 (95% CI = 0.63–1.00)		OR = 0.95 (95% CI = 0.65–1.39)	
Other differences in outcomes	Pneumonia/chronic lung disease 4.7% higher in higher target SaO_2_ group		85–89% group had a decreased rate of O_2_ use at 36 weeks GA (*p* = 0.002)		Risk of necrotising enterocolitis 2.4% higher in 85–89% group (RR = 1.31, 95% CI = 1.01–1.68)		No significant differences in brain injury, necrotising enterocolitis or severed BPD	
**Conclusions**	SaO_2_ has no significant effect on risk of developing ROP SaO_2_ > 96% may have pathological pulmonary effects		Significantly increased mortality among 85–89% group No significant difference in risk of ROP		Significantly increased risk of death in infants with target SaO_2_ < 90% *Recruitment stopped early		No significant differences in mortality or disability (including ROP)	

*OR* Odds ratio (adjusted), *CI* Confidence interval, *RR* Relative risk (adjusted), *BPD* Bronchopulmonary dysplasia

Collectively, these studies demonstrate the balance of supplementary oxygen: higher oxygen saturation targets are associated with ROP and bronchopulmonary dysplasia, whereas a lower target is associated with higher rates of neurological damage, necrotising enterocolitis and death, which caused early termination of the BOOST II trial. Currently, UK guidelines recommend a target oxygen saturation of 91–95% [44]. The 2-phase model of the pathogenesis of ROP may suggest that there is no single target SaO_2_. Rather, when considering ROP in isolation, target SaO_2_ should ideally change over time as the disease moves from its hyperoxic to the hypoxic stage. However, this should be carefully balanced against the systemic implications of changing oxygen saturations and practical challenges of implementation.

#### Pharmacological approaches

Currently, there are two dominant modalities for the treatment of ROP, (1) intravitreal injection of anti-VEGF antibodies and/or (2) laser ablation of the avascular retina. Laser therapy requires prolonged sedation and, even when initiated early as in the Early Treatment of ROP (ETROP) study, cannot prevent retinal detachment in 12% of cases [45]. Thus, increasing focus has been given to a pharmacological approach which would have the benefit of bedside administration, reduced cost and would reduce the burden on specialist ophthalmologists experienced in delivering laser treatments [46].

The antibody bevacizumab binds to all forms of VEGF-A [47]. Intravitreal injection of bevacizumab has been widely used in other vasoproliferative ocular diseases such as age-related macular degeneration, diabetic retinopathy, central and branch retinal vein occlusion in adult patients [48–51]. In addition, intravitreal bevacizumab has been used in children for conditions such as Coats disease and subretinal neovascular membranes. However, its use in all ocular diseases in both adults and children is off-label. Study data shows that, in the UK, Bevacizumab is used by some ophthalmologists to treat A-ROP and posterior type-1 ROP [4].

BEAT-ROP, a multicentre RCT, compared the efficacy of bevacizumab against standard laser treatment, using recurrence of ROP before 54 weeks postmenstrual age as a primary outcome [52]. The authors concluded that the anti-VEGF agent was shown to be effective against stage 3 + disease in zone I, but not in zone II and larger numbers of participants were required to comment on safety. However, these conclusions have been questioned, since using recurrence at 54 weeks as a primary outcome may miss later reactivation of ROP giving a false impression of bevacizumab’s efficacy [53]. The figure of 54 weeks was based on the recurrence of ROP following laser treatment for ROP from the ETROP study but does not take into account the possibility of late reactivation of ROP that is seen in babies treated with anti-VEGF (see below).

A Cochrane Review [54] of anti-VEGF agents for the treatment of ROP included four RCTs (including BEAT-ROP) comparing bevacizumab with laser therapy. The authors concluded that aggregated data suggest bevacizumab as monotherapy does not reduce recurrence or risk of retinal detachment (although, as suggested by BEAT-ROP, treatment for zone I ROP is promising).

Safety concerns have been raised by clinicians regarding the use of anti-VEGF in this age group [53, 55]. Studies in humans show that bevacizumab, when injected intra-ocularly, can escape into the general circulation [56]. Furthermore, primate models demonstrate that the serum concentration of bevacizumab remains raised for up to 8 weeks [57]. The 2018 Cochrane review [54] of anti-VEFG agents concluded that insufficient safety data existed for the recommendation for routine use of bevacizumab. Roohipoor et al. [58] recruited 116 participants, randomised to 0.625 mg bevacizumab or laser therapy. The study found that ROP regressed in all cases of the bevacizumab arm (5 eyes—3.2%—requiring retreatment at a mean of 6.01 weeks after initial treatment) and 97.3% of eyes in the laser therapy arm (*p* = 0.20). Despite follow-up to 90 weeks, only ocular complications were recorded in the bevacizumab arm (one instance of cataract formation).

The dose of bevacizumab used in Roohipoor’s and the BEAT-ROP study was 0.625 mg, half of the adult dose. However, this may be significantly more than required to neutralise VEGF in the vitreous. Further studies looking at lower doses of Bevacizumab ranging from 0.16 to 0.004 mg have shown promising outcomes [59, 60], although there is a likelihood of reactivation occurring sooner with lower doses [61].

An alternative anti-VEGF antibody, Ranibizumab, a human monoclonal antibody fragment derived from the parent antibody Bevacizumab [47] has been licenced and approved for treatment of retinal vasoproliferative disorders in adults and also for ROP by the National Health Service (NHS England) [62] and European Medicines Agency (EMA) [63] following completion of the RAINBOW trial [64]—225 participants, randomized to ranibizumab 0.2 mg, ranibizumab 0.1 mg, and laser photocoagulation therapy. Analysis showed that ranibizumab 0.2 mg may be superior to laser therapy (treatment success odds ratio compared to laser therapy = 2.19. 95% CI 0.99–4.82). Stahl et al. record that “Death, serious and non-serious systemic adverse events, and ocular adverse events were evenly distributed between the three groups” at 24 weeks [64]. One notable advantage that Ranibizumab may have over Bevacizumab is the reduced duration of systemic VEGF suppression [56, 65, 66].

Longer-term follow-up from the CARE-ROP study [67] which compared different doses of ranibizumab (0.2 mg and 0.12 mg) shows no significant difference in ophthalmic and neurodevelopmental outcomes between the treatment arms after 1 and 2 years, respectively.

A third agent, Aflibercept, has the potential to prevent VEGF-driven pathogenesis by binding VEGF-A, VEGF-B as a soluble decoy receptor [68]. Aflibercept is licensed for use in adult retinal vasoproliferative disorders by the US Food and Drug Administration FDA [69] and EMA [70]. The FIREFLEYE study tested Aflibercept in a multicentre, international non-inferiority RCT for treatment of type 1 ROP and A-ROP with an active control comparator group of babies treated with laser therapy [71]. As with the RAINBOW study, the primary outcome was the proportion of infants without active ROP and unfavorable structural outcomes (retinal detachment, macular dragging, macular fold or retrolental opacity) 24 weeks after starting treatment. One hundred eighteen infants were randomized, and 113 were treated in a 2:1 ratio of Aflbercept (0.4 mg) (75 babies) or laser (38 babies). Although treatment success was slightly higher in the Aflibercept-treated arm, the credible interval for the treatment difference was not greater than the prespecified value of − 5% and therefore non-inferiority could not be proven. Further and larger studies of Aflibercept are therefore required to reach any definitive conclusions regarding the comparative effect of Aflibercept to laser in type 1 ROP.

ROP regression following anti-VEGF is a notable for its rapidity. The RAINBOW study documented plus disease regressing significantly faster, at a median time of 4 days (interquartile range (IQR) of 3–8 days) following 0.2 mg of Ranibizumab compared to a median time of 16 days (IQR of 9–22 days) following laser. Similarly, stage 3 was noted to regress at a median of 8 days (IQR 5–17 days) following 0.2 mg of Ranibizumab versus a median of 16 days (IQR 9–29 days) following laser. Indeed, A-ROP was noted to take nearly 3 times longer to regress following laser treatment versus Ranibizumab [72]. This study also showed that incomplete regression of ROP requiring retreatment was more frequently observed in laser-treated eyes with around 22% of eyes requiring further treatment at a median interval of 15 days post-laser compared to 8% of Ranibizumab-treated eyes at a median interval of 21 days after injection [72].

Significant concerns around treatment with anti-VEGF include the prolonged follow-up required to identify re-activation of the disease, that is, a recurrence of acute phase features of ROP, and the management of any persistent avascular retina in the periphery, which arises from interruption of vascular development following intravitreal anti-VEGF administration. Reactivation may occur after complete or incomplete regression of ROP after anti-VEGF injections. It rarely occurs after complete regression of ROP following laser treatment. Reactivation following anti-VEGF treatment may involve, at its mildest, the recurrence of stage 1 demarcation line or more severe features such as stage 3 and/or plus disease. The natural history of such reactivations may not follow through the typical stages of ROP either and may just show features of plus disease associated with fine neovascularisation [34]. Such reactivation has been described more than 12 months after treatment with anti-VEGF (Bevacizumab) [73]. In the RAINBOW study, 31% of infants receiving 0.2 mg of intravitreal Ranibizumab had one or more additional treatments during their follow-up with a median time to first retreatment of 55 days (range 29–111 days) [64]. Ranibizumab may be associated with more rapid reactivation than bevacizumab or aflibercept [74]. Reactivation of ROP following intravitreal anti-VEGF injection may also be seen more in more posterior aggressive diseases or in those injected at a younger post-menstrual age [75]. Very low-dose bevacizumab for treatment of ROP, ranging from 0.002 to 0.016 mg, is also associated with earlier reactivations with a mean of 76.4 days to reactivation compared to low-dose bevacizumab, ranging from 0.031 to 0.25 mg, with a mean of 85.7 days to reactivation [61].

Babies treated with anti-VEGF may never fully vascularise to the peripheral retina leaving a persistent area of avascular retina (PAR) that runs the risk of late reactivation of ROP. This potential for late reactivation in a persistent avascular zone leaves a number of management dilemmas. PAR occurs in untreated, spontaneously regressed ROP as well as in ROP treated with anti-VEGF. Long-term pathological changes within the PAR observed in adult patients with a history of ROP that never reached treatment criteria include atrophic retinal holes, retinal tears, both of which can lead to retinal detachment, tractional retinoschisis (splitting of the retinal layers), vascular anomalies including telangiectatic vessels, vascular leakage and neovascularisation [76]. Similarly fundus fluorescein angiography studies carried out in eyes treated with a history of anti-VEGF injections with PAR demonstrate vascular anomalies such as vascular leakage, shunts, abnormal vessel branching and tangles [77]. There is no consensus regarding the management of such cases in terms of treatment or follow-up regimes. Additionally, in the short to medium term, to continue to undertake regular, serial ROP examinations require parents or guardians to return with their baby for ongoing retinal examinations that may pose practical difficulties, particularly if they have to travel a significant distance. Furthermore, the examinations become increasingly stressful and difficult to carry out in infants with increasing age. Further research is required to answer the questions of whether and when to treat or not.

## Conclusion

ROP is a significant concern in preterm infants and progression to severe disease has a poor visual prognosis. Its pathogenesis can be understood in the context of premature transition from intra- to extra-uterine environments and consequent disruption to oxygen availability in the retina which dictates vascular development via signalling molecules. When considering ROP in isolation, reducing oxygen availability in the retina may help to control this process. However, the implications of this on other developing structures such as the brain and gastrointestinal tract and higher mortality are unacceptable.

Other significant research questions include refining the criteria for identifying babies at risk of serious ROP. The relationship of IGF-1, post-natal weight gain and severe ROP is well established [78] and has encouraged the development of a number of algorithms based on GA, BWT and weight gain and ethnicity [79, 80]. Implementation of refined and validated screening algorithms may reduce the overall burden of screening.

The increasing uptake of digital retinal imaging and subsequent capability of image analysis also lends itself to the development of artificial intelligence and deep learning algorithms to assist clinicians in identifying critical features of ROP, such as plus disease versus no plus or pre-plus disease, vascular severity scores or stage of ROP. Many are showing promise but as yet are not reliable enough to entirely replace the experienced clinician [81].

Interventions such as laser ablation of the avascular retina persist, but more recently anti-VEGF agents offer a more targeted approach. Future optimal dosage studies may eradicate long-term wider safety fears and elucidate optimum dosages or therapeutic agents specific to the stage and zone of ROP. Further studies may also address the problem of management of the peripheral avascular retina following anti-VEGF treatment. Going forward, alternative ways of targeting VEGF, such as the use of the beta-blocker, propranolol [82] and the targeting of other pathological pathways that lead to ROP, such as LCPUFAs and IGF-1 may warrant further investigation.

## Data Availability

This review used published literature, accessed through the University of Birmingham Library.

## Abbreviations

A-ROP: Aggressive Retinopathy of Prematurity
BEAT-ROP: Bevacizimab Eliminates the Angiogenic Threat of Retinopathy of Prematurity
BOOST II: Benefits of Oxygen Saturation Targeting
BW: Birth weight
CARE-ROP: Comparing Alternative Ranibizumab Dosages for Safety and Efficacy in Retinopathy of Prematurity
COT: Canadian Oxygen Trial
ELGAN: Extremely low gestational age newborns
EMA: European Medicines Agency
ETROP: Early Treatment of Retinopathy of Prematurity
EPO: Erythropoietin
FDA: Food and Drug Administration (US)
FIREFLEYE: Aflibercept for Retinopathy of Prematurity—Intravitreal Injection Versus Laser Therapy (FIREFLEYE)
GA: Gestational age
HIFs: Hypoxia-induced factors
ICROP3: International Classification of Retinopathy of Prematurity 3
IGF-1: Insulin-like growth factor-1
LCPUFAs: Long-chain polyunsaturated fatty acids
NHS: National Health Service (UK)
PAR: Persistent avascular retina
PMA: Postmenstrual age
PO2: Partial pressure of oxygen
RAINBOW: RAnibizumab Compared with Laser Therapy for the Treatment of INfants BOrn Prematurely With Retinopathy of Prematurity
RCTs: Randomized control trials
ROP: Retinopathy of prematurity
SaO2: Oxygen saturation
STOP-ROP: Supplemental Therapeutic Oxygen for Prethreshold Retinopathy of Prematurity
SUPPORT: Surfactant, Positive Pressure, and Pulse Oximetry Randomized Trial
VEGF: Vascular endothelial growth factor
VEGFR: Vascular endothelial growth factor receptor
